# Optshrink LR + S: accelerated fMRI reconstruction using non-convex optimal singular value shrinkage

**DOI:** 10.1007/s40708-016-0059-x

**Published:** 2017-01-10

**Authors:** Priya Aggarwal, Parth Shrivastava, Tanay Kabra, Anubha Gupta

**Affiliations:** SBILab, Department of Electronics and Communication Engineering, IIIT-Delhi, New Delhi, India

**Keywords:** Accelerated functional MRI, Low-rank recovery, Sparse recovery, Compressed sensing, *k–t* acceleration, Undersampling

## Abstract

This paper presents a new accelerated fMRI reconstruction method, namely, *OptShrink LR + S* method that reconstructs undersampled fMRI data using a linear combination of low-rank and sparse components. The low-rank component has been estimated using non-convex optimal singular value shrinkage algorithm, while the sparse component has been estimated using convex *l* ^1^ minimization. The performance of the proposed method is compared with the existing state-of-the-art algorithms on real fMRI dataset. The proposed *OptShrink LR + S* method yields good qualitative and quantitative results.

## Introduction

Functional magnetic resonance imaging (fMRI) is one of the most significant noninvasive and non-ionizing diagnostic imaging modality [1, 2]. It measures blood oxygenated level dependent (BOLD) signal for localizing brain activity [3]. However, despite the advancements in fMRI scanners, one of the biggest limitations of fMRI modality is slow imaging compared to the other medical imaging modalities [4].

Conventionally, parallel imaging techniques such as Sensitivity Encoding (SENSE) [5, 6], Generalized Autocalibrating Partially Parallel Acquisitions (GRAPPA) [7, 8], and Simultaneous Acquisition of Spatial Harmonics (SMASH) [9] are used to accelerate magnetic resonance imaging (MRI). Here, the basic principle involves use of multiple receiver coils with complementary sensitivity information. SENSE, GRAPPA, or SMASH reconstruct MRI images from multiple *k*-space undersampled images acquired on different coils. For the case of fMRI, only *k*–*t* GRAPPA is able to accurately reconstruct fMRI images [10]. However, this method introduces strong temporal autocorrelation in the data that limits the extent of undersampling of fMRI data [10].

Apart from parallel imaging, compressed sensing (CS)-based fMRI reconstruction is another attractive method of fMRI acceleration [11–17]. Similar to parallel imaging, less data are acquired in the *k*-space (spatial frequency domain) in CS resulting in accelerated fMRI data acquisition. However, unlike GRAPPA and SENSE that does not exploit information contained across time frames, CS exploits information across time frames leading to sparse representation and hence provides good reconstruction quality. Reconstruction of full fMRI data from this less or undersampled data requires efficient reconstruction algorithm. Researchers have proposed various methods for efficient reconstruction from undersampled *k*-space measurements [11–17]. These methods largely rely on compressive sensing and reconstruct data using an optimization framework under certain constraints. Often, fMRI data are assumed to be sparse in some transform domain. Theoretical studies have shown that it is possible to recover sparse signals by *l* ^1^ norm minimization [18]. For example, in [13], undersampled fMRI data are reconstructed using CS with sparsity of fMRI data in the wavelet domain, wherein orthogonal Daubechies wavelet is used as the sparsifying basis. This is to note that CS-based sparse recovery methods are being used extensively in many applications including other medical imaging modalities [19, 20] and in videos [21, 22].

In general, fMRI data matrix ${\mathbf{X }}$, i.e., one fMRI slice data stacked over time, is observed to be low rank. Hence, low-rank constraint can be imposed in the CS-based optimization framework to recover fMRI data slice by slice. Recently, *k*–*t* FASTER method has been proposed on similar lines that recovers fMRI signal via hard thresholding of singular values of low-rank data matrix **X** in the CS framework [11].

In [15], a new LR + S method had been proposed to reconstruct fMRI data that uses a linear combination of low-rank and sparse components, i.e.,

1 $$\begin{aligned} {\mathbf{X }}={\mathbf{L}} (\hbox {low rank})+{\mathbf{S }}(\hbox {sparse}). \end{aligned}$$

This decomposition of data into low-rank and sparse component is popularly known as robust principal component analysis (RPCA) in the literature [23, 24]. In RPCA, convex optimization-based approaches are used to recover low-rank and sparse components from matrix ${\mathbf{X }}$. In [15], fMRI reconstruction is solved via iterative estimation of ${\mathbf{L }}$ and ${\mathbf{S}}$ components using convex optimization-based approaches. In noise-free scenarios, convex approaches may still provide reasonable solution for non-convex problems [25]. However, in noisy settings, such as in fMRI with low signal-to-noise ratio (SNR), convex optimization-based methods may not provide optimum or close to optimum solution.

For quality accelerated fMRI reconstruction in noisy settings, improved low-rank matrix and sparse matrix estimation are necessary. There has been a great interest to recover low-rank matrix from noisy measurements in various fields such as statistical signal processing [26–28], machine learning [29], and estimation and classification problems [30]. This motivates us to explore an improved method of accelerated fMRI reconstruction that can recover denoised low-rank matrix and sparse component from the undersampled *k*-space data.

We use optimal singular value shrinkage denoising algorithm (OptShrink), a data-driven method, recently used for denoising of low-rank matrix [31]. We call the proposed method as *Optshrink LR + S* method. In [31], OptShrink has been shown to have improved performance over singular value thresholding (SVT) in the recovery of data with missing entries. The OptShrink method requires noisy low-rank matrix and its rank estimate as input and provides denoised low-rank matrix estimate.

The proposed *Optshrink LR + S* fMRI reconstruction method is compared with other offline fMRI reconstruction methods such as direct inverse Fourier transform (IFT), LR + S [15], and CS with wavelet sparsity [13] methods. We compare reconstruction results using different methods at both the subject- and group level at different acceleration factors. Our proposed *OptShrink LR + S* method reconstructs fMRI data with greater accuracy compared to other methods even at lower sampling ratios.

The rest of this paper is organized as follows. Section 2 discusses fMRI reconstruction problem and presents the proposed *Optshrink LR + S* reconstruction method. In Sect. 3, simulation results using *Optshrink LR + S* and some of the existing methods are presented on real fMRI data. Conclusions are presented in the last section.

## Materials and methods

In this section, we present the mathematical formulation of fMRI reconstruction problem followed by details of the proposed *Optshrink LR + S* fMRI reconstruction method and description of the fMRI dataset used in simulations.

### Problem formulation

The functional MRI imaging involves acquisition of contiguous brain slices over a number of time points. For each individual brain slice, Casorati matrix ${\mathbf{X }}\in {\mathbb {R}}^{n\times T}$ is formed by stacking one brain slice over all time points [32], i.e., ${\mathbf{X }}=\left\{ {\mathbf{x }}_{i},i=1,\ldots ,T \right\}$, where *T* is the number of time points and *n* is the number of voxels in one brain slice. Hence, each column ${\mathbf{x }}_{i}$ of ${\mathbf{X }}$ corresponds to data of a particular brain slice captured at one time point. Let us denote the undersampled *k*-space fMRI data of one brain slice captured over time by the matrix ${\mathbf{Y }}$.

The relationship between undersampled *k*-space data ${\mathbf{Y }}$ and ${\mathbf{X}}$ is as follows:

2 $$\begin{aligned} {\mathbf{Y }}={\mathbf{\Phi }} {\mathbf{FX }}+{\mathbf {\xi }}, \end{aligned}$$

where ${\mathbf{F }}$ denotes the two-dimensional (2-D) Fourier transform operator, ${\mathbf {\Phi }}$ is the measurement matrix that detects or captures fewer *k*-space measurements, and $\xi \in {\mathbb {R}}^{n\times T}$ denotes the measurement noise. In fMRI reconstruction problem, matrix **X** is required to be recovered from the undersampled *k*-space fMRI data measurements in matrix **Y**.

### Reconstruction using low-rank plus sparse decomposition

In this paper, we are interested in accelerated fMRI data reconstruction using low-rank plus sparse decomposition. Hence, in this subsection we first elucidate low-rank plus sparse reconstruction problem.

Consider matrix ${\mathbf{X }}$ as the superposition of low-rank matrix ${\mathbf{L }}$ with rank *m* and sparse matrix ${\mathbf{S }}$ with sparsity *s*. Hence, matrix ${\mathbf{X }}$ can be denoted as ${\mathbf{X }}:={\mathbf{L}} +{\mathbf{S }}\in {\mathbb {R}}^{n\times T}$, where matrices ${\mathbf{L }}$ and ${\mathbf{S}}$ are required to be recovered, given a set of undersampled measurements ${\mathbf{Y} }$ and the corresponding measurement matrix ${\mathbf {\Phi }}$. The optimization problem for identifying matrix $\hat{{\mathbf{L }}}\in {\mathbb {R}}^{n\times T}$ and matrix $\hat{{\mathbf{S }}}\in {\mathbb {R}}^{n\times T}$ from ${\mathbf{Y }}$ and ${\mathbf {\Phi }}$ can be written as:

3 $$\begin{aligned} \hat{\mathbf {L}},\hat{\mathbf {S}}=\underset{{\mathbf {L,S}}:~{\mathrm{rank}}({\mathbf {L}})\le m,\parallel {\mathbf {S}}\parallel _{0}\le s}{\hbox {arg min}}\parallel {\mathbf {Y}}-{\mathbf {\Phi }} {\mathbf {F(L+S)}}\parallel _{F}, \end{aligned}$$

where the Frobenius norm $\parallel {\mathbf {Y}}-{\mathbf {\Phi }} {\mathbf {F(L+S)}}\parallel _{F}$ is defined as $\parallel {\mathbf {Y}}-{\mathbf {\Phi }} {\mathbf {F(L+S)}}\parallel _{F}=\hbox {Tr}[({\mathbf {Y}}-{\mathbf {\Phi }} {\mathbf {F(L+S)}})^{\mathrm{T}}({\mathbf {Y}}-{\mathbf {\Phi }} {\mathbf {F(L+S))]}}$, $\hbox {Tr}(\cdot )$ denotes trace of matrix, and $(\cdot )^{\mathrm{T}}$ denotes transpose. Problem in (3) can be solved iteratively when there is incoherence between low-rank matrix ${\mathbf{L }}$ and sparse matrix ${\mathbf{S }}$ matrices [15, 23, 24]. It is observed that incoherence is guaranteed when low-rank matrix is not sparse and sparse matrix is not low rank [15, 24].

### Proposed *Optshrink LR + S* method

In this subsection, we explain the proposed Optshrink LR + S reconstruction method wherein the problem in (3) is solved by breaking it into two subproblems of estimating ${\mathbf{L }}$ and ${\mathbf{S}}$ as described below.

#### **S** subproblem

In (3), $\parallel {\mathbf{S }} \parallel _{0}$ denotes $l^{0}$ norm that is equal to the number of nonzero values (= *s*) in matrix **S**. $l^{0}$ norm is a non-deterministic polynomial (NP) hard problem [33]. Thus, $l^{1}$ norm is generally used as the closest convex surrogate of $l^{0}$ norm [18, 34]. $l^{1}$ norm is defined as absolute sum of values in matrix **S** and is used to obtain sparse solution [18, 34]. Generally, soft thresholding (ST) is used to solve $l^{1}$ norm penalty on **S** defined as:

4 $$\begin{aligned} \hat{\mathbf {S}} =\hbox {Soft}({\mathbf {S}},\lambda _{1}):=\hbox {sgn}({\mathbf {S}})\otimes \max \left\{ 0,\left| {\mathbf {S}} \right| -\lambda _{1} \right\} , \end{aligned}$$

where $\otimes$ denotes component-wise product and $\lambda _{1}$ is the soft thresholding regularization parameter on ${\mathbf {S}}$. Recently in [15], sparse matrix is recovered using ST on ${\mathbf{S }}$. We use similar approach of ST in this work to solve for sparsity on ${\mathbf{S }}$.

#### **L** subproblem

Low-rank matrix recovery is ill-posed and NP hard [35]. One of the methods to solve this problem is via convex optimization using nuclear norm minimization [35]. Nuclear norm minimization implies $l^{1}$ penalty on singular values of matrix **L** that supports matrix **L** to be low rank. Global minimum of convex nuclear norm minimization is obtained by soft thresholding on singular values, known as singular value thresholding (SVT) [36].

To understand this, consider $n\times T$ noisy low-rank matrix:

5 $$\begin{aligned} {\tilde{\mathbf{L}}}={\mathbf {L}}+{\mathbf {\delta }}, \end{aligned}$$

where ${\mathbf {L}}$ is the noise-free low-rank matrix and ${\mathbf {\delta }}$ is a random noise matrix. Here, the goal is to estimate non-noisy low-rank matrix ${\mathbf{L }}$ from noisy matrix ${\tilde{\mathbf{L}}}$.

Let singular value decomposition (SVD) of matrix ${\tilde{\mathbf{L}}}$ is $\sum _{i=1}^{q}\sigma _{i}{\mathbf{u }}_{i} {\mathbf{v}} _{i}^{\mathrm{H}}$, where $\sigma _{i}, {\mathbf{u }}_{i}$ and ${\mathbf{v }}_{i}$ are the singular values, left singular vectors, and right singular vectors, respectively; $q=\hbox {min}(n,T)$ denotes the rank of ${\tilde{\mathbf{L}}}$ and $(\cdot )^{\mathrm{H}}$ denotes the conjugate transpose. Convex nuclear norm solution of (5) can recover non-noisy low-rank matrix via SVT [36] as:

6 $$\begin{aligned} \hat{{\mathbf{L }}}=\hbox {SVT}({\tilde{\mathbf{L}}};\lambda _{2} )=\sum _{i=1}^{q}\hbox {Soft}(\sigma _{i},\lambda _{2}){\mathbf{u} }_{i} {\mathbf{v}} _{i}^{\mathrm{H}}, \end{aligned}$$

where definition of ‘Soft’ is same as defined in (4) and $\lambda _{2}$ in (6) is the regularization parameter.

Recently, in [15], low-rank matrix is recovered using SVT, where noisy input low-rank matrix is initialized from the previous iteration. The key idea behind SVT is to shrink nonsignificant singular values toward zero while keeping the singular vectors unchanged. However, nuclear norm minimization is an over-relaxing recovery solution of low-rank matrix [37].

In this paper, we propose to estimate non-noisy low-rank matrix or denoised approximation for the low-rank matrix in (5) that will provide an overall improved performance of fMRI signal reconstruction using low-rank plus sparse decomposition. In [31], best approximate noise-free low-rank matrix is obtained by optimal weighted combination of left and right singular vectors of input noisy matrix ${\tilde{\mathbf{L}}}$ in (5). Let us assume that low-rank matrix **L** has rank *m* [refer to (5)] and is given as

7 $$\begin{aligned} {\mathbf{L }}=\sum _{i=1}^{m}w_{i}{\mathbf{u }}_{i} {\mathbf{v}} _{i}^{\mathrm{H}}, \end{aligned}$$

where ${\mathbf{u }}_{i}$ and ${\mathbf{v }}_{i}$ are the left and right singular vectors of noisy matrix ${\tilde{\mathbf{L}}}$ and $w_{i}$ are unknown singular values. In order to recover ${\mathbf {L}}$ from the noisy matrix ${\tilde{\mathbf{L}}}$ in (5), the problem is formulated as:

8 $$\begin{aligned} \hat{{\mathbf{L }}}=\underset{{\mathbf {L}}}{\hbox {arg min}}\left\| {\tilde{\mathbf{L}}}-{\mathbf {L}} \right\| _{F} \quad \text {with}\quad \hbox {rank}({\mathbf {L}})=m. \end{aligned}$$

Using (7), we can rewrite (8) as:

9 $$\begin{aligned} {\mathbf {w}}^{opt}=\underset{\left\| {\mathbf {w}} \right\| _{l_{0}}=m}{\hbox {arg min}}\left\| {\tilde{\mathbf{L}}}-\sum _{i=1}^{m}w _{i}{\mathbf{u }}_{i} {\mathbf{v}} _{i}^{\mathrm{H}}\right\| _{F}. \end{aligned}$$

where, $l^{0}$ norm on ${\mathbf{w }}$ in (9) signifies number of nonzero singular values equal to the rank *m*. In the above equation, singular vectors ${\mathbf{u }}_{i}$ and ${\mathbf{v }}_{i}$ are known and estimated using SVD of input matrix ${\tilde{\mathbf{L}}}$. The closed form solution of singular values in (9) for every $1\le i\le m$ is expressed as [31]:

10 $$\begin{aligned} w_{i}^{\mathrm{opt}}=-2~\frac{D(\sigma _{i};{\mathbf {\Sigma }})}{D^{'}(\sigma _{i};{\mathbf {\Sigma }} )}, \end{aligned}$$

where ${\mathbf {\Sigma }}\in {\mathbb {R}}^{n\times T}$ and is equal to $\text {diag}(\sigma _{m+1},\ldots \sigma _{q})$, $q=\min (n,T)$, $\sigma _{i}$ denotes the *i*th singular value of ${\tilde{\mathbf{L}}}$, $\hbox {Tr}(\cdot )$ is equal to the trace of a matrix, and **I** is the identity matrix. $D(\cdot )$ is the *D*-transform which is defined as

11 $$\begin{aligned} D(\sigma _{i};{\mathbf {\Sigma }}) & := \frac{1}{n}\text {Tr}\left( \sigma _{i}\left( \sigma _{i}^{2}{\mathbf{I }}-{\mathbf {\Sigma }} {\mathbf {\Sigma }} ^{\mathrm{H}}\right) ^{-1}\right) \nonumber \\&\quad \times \frac{1}{T}\text {Tr}\left( \sigma _{i}\left( \sigma _{i}^{2}{\mathbf{I }}- {\mathbf {\Sigma }} ^{\mathrm{H}}{\mathbf {\Sigma }}\right) ^{-1}\right) , \end{aligned}$$

and $D^{'}(\cdot )$ is defined as

12 $$\begin{aligned} D^{'}\left( \sigma _{i};{\mathbf {\Sigma }}\right) & := \frac{1}{n}\text {Tr}\left( \sigma _{i}\left( \sigma _{i}^{2}{\mathbf{I }}-{\mathbf {\Sigma }} {\mathbf {\Sigma }} ^{\mathrm{H}}\right) ^{-1}\right) \times \frac{1}{T}\text {Tr}\left( -2\sigma _{i}^{2}\left( \sigma _{i}^{2}{\mathbf{I }} \right. \right. \nonumber \\&\quad \left. \left. - {\mathbf {\Sigma }} ^{\mathrm{H}}{\mathbf {\Sigma }}\right) ^{-2}+\left( \sigma _{i}^{2}{\mathbf{I }}-{\mathbf {\Sigma }}^{\mathrm{H}} {\mathbf {\Sigma }} \right) ^{-1}\right) +\frac{1}{T}\text {Tr}\left( \sigma _{i}\left( \sigma _{i}^{2}{\mathbf{I }}-{\mathbf {\Sigma }}^{\mathrm{H}} {\mathbf {\Sigma }} \right) ^{-1}\right. \nonumber \\&\quad \left. \times \frac{1}{n}\text {Tr}\left( -2\sigma _{i}^{2}\left( \sigma _{i}^{2}{\mathbf{I }}- {\mathbf {\Sigma }}{\mathbf {\Sigma }} ^{\mathrm{H}}\right) ^{-2}+\left( \sigma _{i}^{2}{\mathbf{I }}-{\mathbf {\Sigma }} {\mathbf {\Sigma }} ^{\mathrm{H}}\right) ^{-1}\right) \right) , \end{aligned}$$

This algorithm is named as Optshrink [31]. OptShrink is a data-driven method, recently used for denoising of low-rank matrix in an application of signal recovery in missing data. It considers noisy low-rank matrix and its rank estimate [= *m* in (7)] as input, and provides denoised estimate of the low-rank matrix as the output. It is a non-convex solution that does weighing of singular vectors. It shrinks the corresponding singular values using truncated singular value decomposition (TSVD) and hence is called non-convex optimal SVT. This algorithm works better than SVT [31].

Also, in [31], it has been shown that the solution of Optshrink is quite robust to input rank specification, and hence a rough estimate of rank [= *m* in (7)] at the input is sufficient. Another advantage of Optshrink is that there is no need to specify shrinkage parameter as is required in SVT [refer to $\lambda _{2}$ in (6)]. In SVT, we need to tune $\lambda _{2}$ for every dataset. It has been observed that Optshrink always outperforms SVT in the estimation of low-rank matrix.

In this paper, we propose to apply OptShrink for low-rank matrix estimation in fMRI reconstruction using low-rank plus sparse decomposition. fMRI data inherently have low signal-to-noise ratio (SNR). Hence, fMRI reconstruction with OptShrink for denoised low-rank matrix estimation should outperform existing low-rank plus sparse fMRI reconstruction method [15].

#### Overall solution of (3) using Optshrink LR + S

Finally, Eq. (3) is solved iteratively using the proposed *Optshrink LR + S* method as below:

13 $$\begin{aligned} \hat{\mathbf {S}}^{j}& = \hbox{Soft}_{\lambda_{1}}{\mathbf {\Psi }}(\hat{\mathbf{X}}^{j-1}-\hat{\mathbf{L}}^{j-1})\nonumber \\ \hat{\mathbf {L}}^{j} & = \hbox{Optshrink}_{\lambda _{2}}(\hat{\mathbf{X}}^{j-1}-\hat{\mathbf{S}}^{j-1})\nonumber \\ \hat{\mathbf{X}}^{j} & = \hat{\mathbf{L}}^{j}+\hat{\mathbf{S}}^{j}-{\mathbf{A}}^{\mathrm{T}}({\mathbf{A}} (\hat{\mathbf{L}}^{j}+\hat{\mathbf{S}}^{j})-{\mathbf{Y}}), \end{aligned}$$

where ${\mathbf{A }}={\mathbf {\Phi }}{\mathbf{F }}$ in (13) and *j* denotes an iteration number. Here, ST is used to recover sparse matrix **S** as explained in Sect. 2.3.1 and Optshrink algorithm is used to solve for low-rank matrix **L** as explained in Sect. 2.3.2. Voxel time series are observed to be sparse in the Fourier domain. Hence, variation along rows of matrix ${\mathbf{X }}$ is assumed to be sparse in the Fourier domain. ${\mathbf {\Psi }}$ in Algorithm 1 is the sparsifying matrix for the Fourier domain where Fourier transform is to be taken along the rows. Solution is updated at each iteration *j*. Algorithm 1 presents the pseudo-code of *Optshrink LR + S* method.

Please note that low-rank component represents the background information that is highly correlated across data captured at different time points and sparse component represents the dynamic and uncorrelated counterpart.
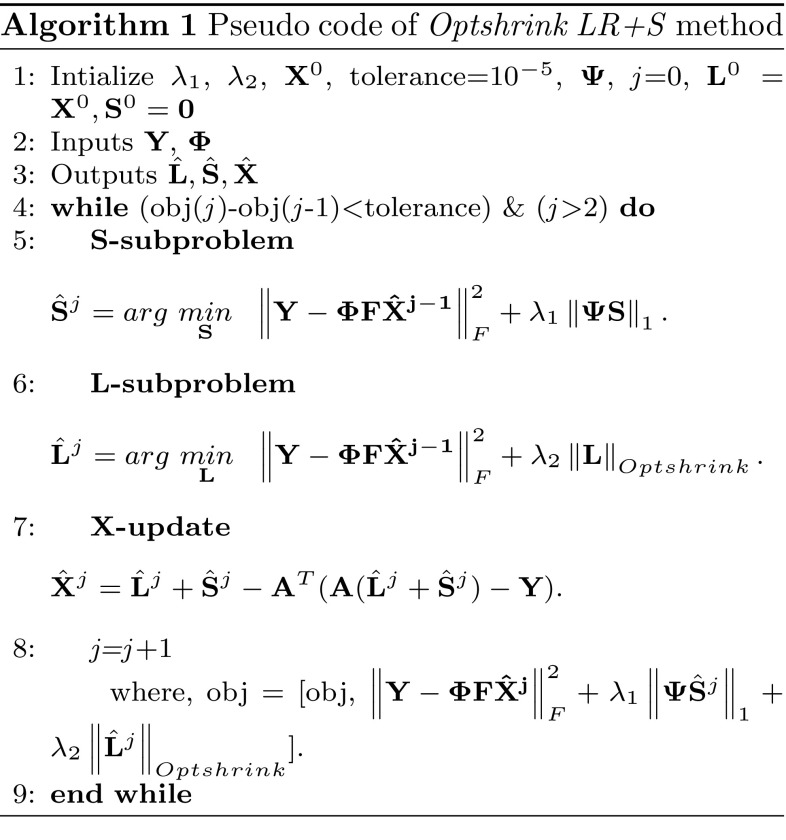


### Dataset description

To assess the performance of reconstruction methods, we have used two fMRI datasets in this paper: (1) Task-based fMRI dataset with false belief task (OpenfMRI publicly available dataset)^1^ and (2) Resting-state Baltimore fMRI dataset (1000 functional Connectomes Project Data).^2^

#### Task-based dataset

This dataset consists of acquisition of 36 axial interleaved brain slices with dimensions 72x72 at each time point with echo time (TE) equal to 35 ms and repetition time (TR) equal to 2 s [38]. These data are collected over 179 time points, resulting in the matrix ${\mathbf{X }}$ of size $5184\times 179$ for one brain slice. During the false belief experiment, the subject had to answer questions about stories that referred either to a person’s false belief (mental trials) or to outdated physical representations such as an old photograph. For more details on this dataset, please refer to [38].

#### Resting-state dataset

These data are publicly available as part of the 1000 Functional Connectomes Project. This is a collection of resting-state fMRI dataset from a number of laboratories around the world. We use Baltimore resting fMRI data. This dataset consists of 23 subjects resting-state fMRI data, aged between 20 and 40 years of age, acquired while subjects’ eyes were open and fixated on a screen. The repetition time (TR) is 2.5 s, size of a brain volume at one time point is $96\times 96\times 47$, and the total no. of time points over which data are captured is 123.

## Simulation results

**Fig. 1:**
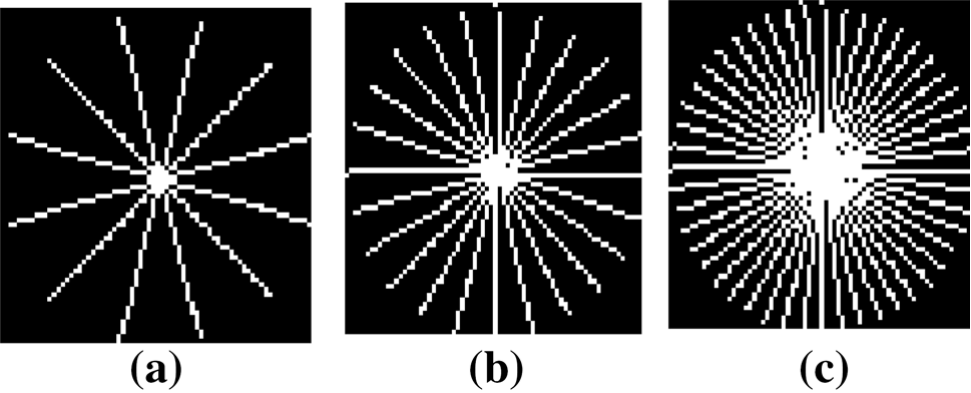
Radial sampling pattern on one slice: **a** 6 radial lines (12.856 acceleration factor); **b** 12 radial lines (6.065 acceleration factor); **c** 24 radial lines (3.495 acceleration factor)

Since both resting-state fMRI dataset (Baltimore dataset) and task-based fMRI dataset (false belief fMRI dataset) are fully sampled, we simulated undersampled *k*-space data ${\mathbf{Y }}$ in (2) by computing the Fourier transform of Casorati matrix ${\mathbf{X }}$ and then, retrospectively undersampling in the *k*-space using measurement matrix ${\mathbf {\Phi }}$. This measurement matrix is generated using radial sampling patterns. We used three radial sampling patterns with different acceleration factors for testing reconstruction performance: 6 radial lines, 12 radial lines, and 24 radial lines as described in [39]. Radial sampling pattern is chosen because this is one of the fastest *k*-space sampling methods in real-time application [39]. Figure 1 shows these radial sampling measurement patterns. These radial sampling patterns sample more data in the low-frequency region compared to the high-frequency region.

This is to note that we have illustrated undersampling of fMRI data by retrospective sampling on the Cartesian grid because it allows sampling patterns to maintain incoherency among the columns of matrix X [39–41]. However, radial-Cartesian sampling grid is more realistic from the point of view of actual data acquisition [10, 42]. Similarly in [12], variable density spiral sampling pattern has been used in MRI scanner and is shown to be robust against motion, off resonance, and gradients artifacts in compressed sensing fMRI application. However, our work is focused on development of robust reconstruction algorithm. This is to note that the proposed Optshrink LR + S method reconstructs fMRI data as a superposition of low-rank and sparse matrix, where the low-rank component represents background information that is highly correlated across data captured at different time points and sparse component represents the dynamic and uncorrelated part. Since these assumptions are characteristic of fMRI data, they will remain valid irrespective of the sampling strategy used. Hence, although the proposed work is general and can be used with any sampling pattern *provided sampling incoherence is maintained*, we project the use of realistic sampling patterns as the future work.

Data obtained from the database are called as original data in the manuscript. *k*-space data are acquired by considering 2-D Fourier transform of this original data. Since the original data are real and are provided without any phase information, we only considered the magnitude part of the reconstructed data. Thus, no assumption is made about the phase part of the data. This is to clarify that this is a standard method of testing newer MRI/fMRI reconstruction algorithms via simulation results.

### Comparison with different methods

In this section, we provide results on fMRI reconstruction from undersampled *k*-space fMRI data using the proposed *Optshrink LR + S* method, existing LR + S method [15], direct inverse Fourier transform-based reconstruction, and reconstruction using CS with wavelet sparsity [13]. Below, we present a brief overview of each of these existing reconstruction methods used.

#### Low-rank plus sparse (LR + S) method [15]

This method reconstructs fMRI data using superposition of low-rank and sparse matrix components [15], and hence, the optimization problem is:

14 $$\begin{aligned} \hat{{\mathbf {L}}},\hat{\mathbf {S}}=\underset{{\mathbf {L,S}}}{\hbox {arg min}} \left\| {\mathbf {Y}}-{\mathbf {\Phi }} {\mathbf {F(L+S)}} \right\| _{F}^{2}+\lambda _{1}\left\| {\mathbf {\Psi }}{\mathbf {S}} \right\| _{1}+\lambda _{2}\left\| {\mathbf {L}} \right\| _{*}. \end{aligned}$$

We empirically selected $\lambda _{1}=2$ and $\lambda _{2}=200$ in the above Eq. (14) using the *L*-curve method [43]. Minimum normalized mean square error (NMSE) is obtained in the *L*-curve at the above chosen $\lambda$s for the existing LR + S method. This is to note that we used same values of $\lambda$s in the proposed Optshrink LR + S method. Thus, the values of $\lambda$s are optimally selected for the existing LR + S method and not for the proposed Optshrink method for presenting the comparative results.

#### Direct IFT

This method computes 2-D inverse Fourier transform (IFT) of given *k*-space fMRI data ${\mathbf{Y }}$ and reconstructs ${\mathbf{X }}$ as shown below:

15 $$\begin{aligned} \hat{{\mathbf {X}}}=\hbox {IFT}({\mathbf {Y}}). \end{aligned}$$

#### CS with wavelet sparsity (CSWD) [13]

Wavelet sparsity assumes signal to be sparse in the wavelet domain [13], and hence, the optimization problem is:

16 $$\begin{aligned} \hat{{\mathbf {X}}}=\underset{{\mathbf {X}}}{\hbox {arg min}} \left\| {\mathbf {Y}}-{\mathbf {\Phi }} {\mathbf {FX}} \right\| _{F}^{2}+\lambda _{3}\left\| {\mathbf {W}}{\mathbf {X}} \right\| _{1 }, \end{aligned}$$

where ${\mathbf {W}}$ is a wavelet matrix operator. We used Daubechies’ orthogonal wavelet ‘db4’ (filter lengths 8) with three-level decomposition as the sparsifying basis to exploit wavelet sparsity as used in [13]. This method requires one parameter $\lambda _{3}$ to be specified as shown in (16). In [44], $\lambda _{3}$ is restricted to satisfy the below condition:

17 $$\begin{aligned} \lambda _{3} < \max \left( {\mathbf {\Phi }}^{\mathrm{T}}\left( \hbox {IFT}\left( {\mathbf{Y }}\right) \right) \right) . \end{aligned}$$

In order to meet the above condition, we chose

18 $$\begin{aligned} \lambda _{3} =0.009\times \max \left( {\mathbf {\Phi }}^{\mathrm{T}}\left( \hbox {IFT}\left( {\mathbf{Y }}\right) \right) \right) \end{aligned}$$

that meets (17).

**Fig. 2:**
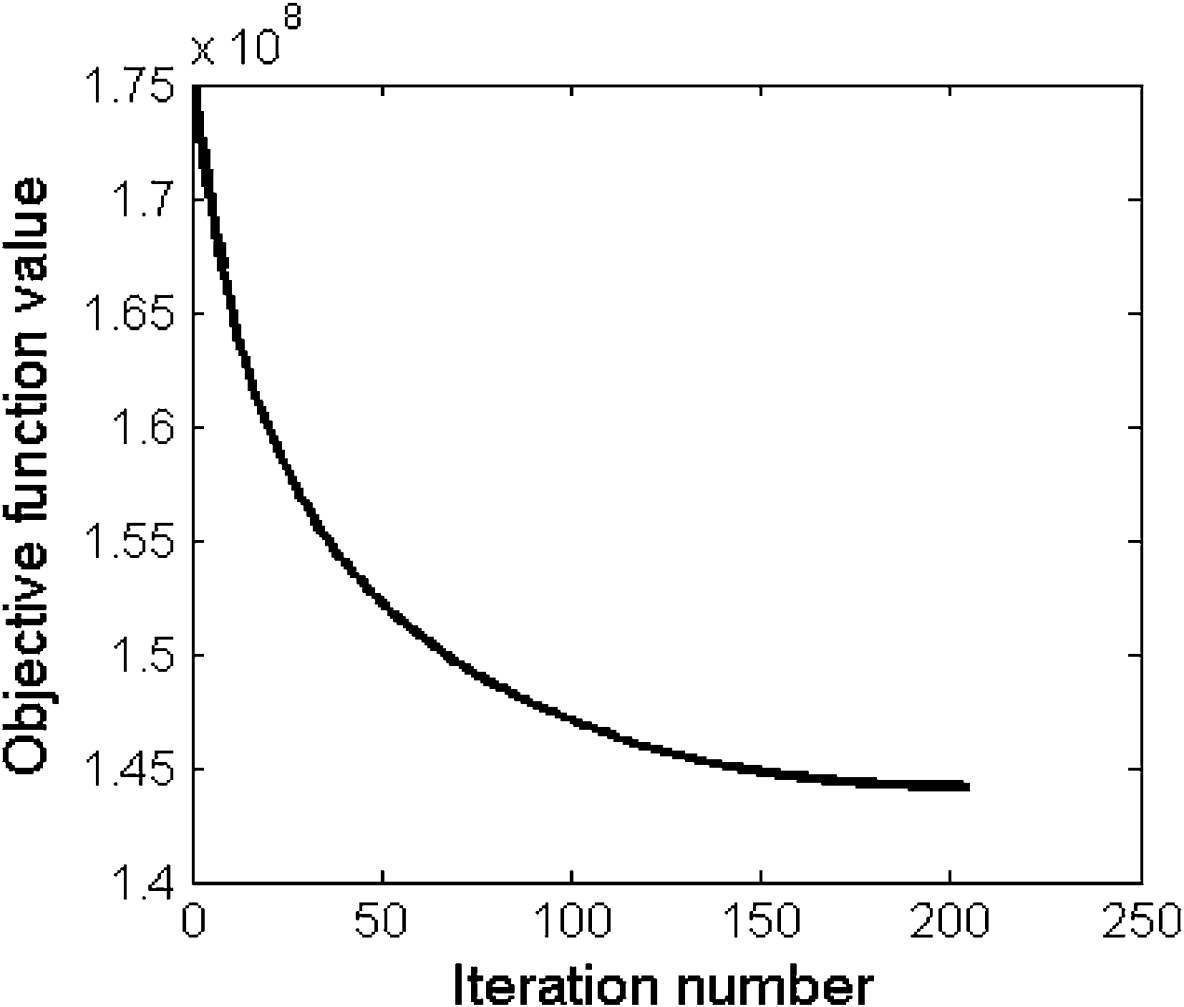
Objective function value versus number of iterations

For all reconstruction methods, we set the maximum number of iterations equal to 500 and use the following convergence criterion: $\hbox {objective function value} (\hbox {end})-\hbox {objective function value} (\hbox {end}-1)<10^{-5}$, also specified in Algorithm 1. Figure 2 shows objective function value versus number of iterations on one subject of the task-based dataset with the proposed Optshrink LR + S method. From this figure, we observe that the objective function converges monotonically. We observed the same trend with every data, and hence, we may safely state that the proposed Optshrink LR + S method convergences to provide solution.

**Fig. 3:**
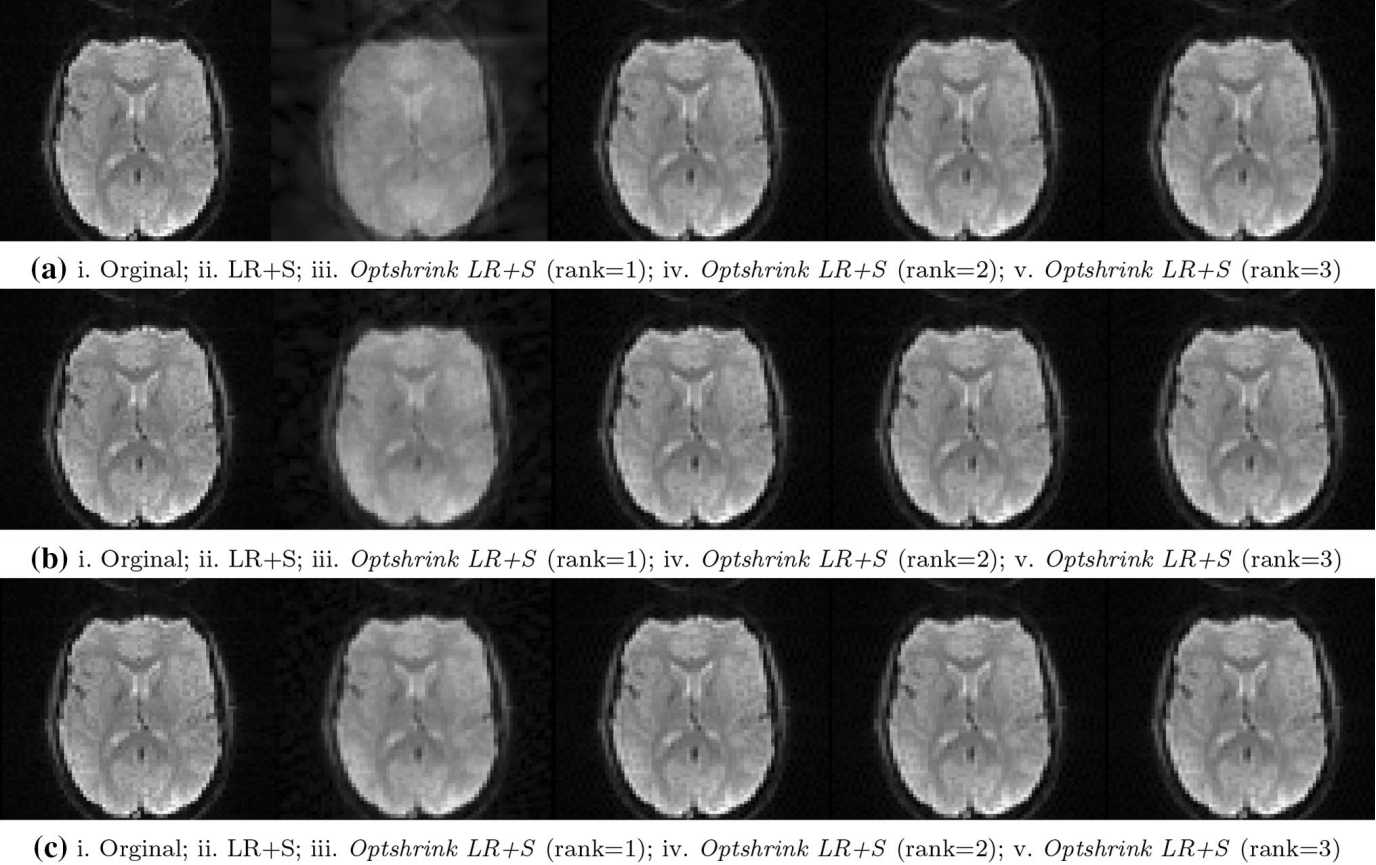
Task-based fMRI data–original and reconstructed slice no. 18 [*left to right* Original; LR + S; *Optshrink LR + S* (rank = 1); *Optshrink LR + S* (rank = 2); *Optshrink LR + S* (rank = 3)]: **a** 6 radial lines (time point 100); **b** 12 radial lines (time point 100); **c** 24 radial lines (time point 100)

Figure 3 shows one of the reconstructed brain slices of task-based fMRI dataset (false belief task) using the proposed *Optshrink LR + S* and the existing LR + S [15] method. Reconstructed data are visually shown at different radial lines in Fig. 3a, b, c corresponding to the middle slice (slice number 18 of 36 no. of total slices) captured at the 100th time point.

**Fig. 4:**
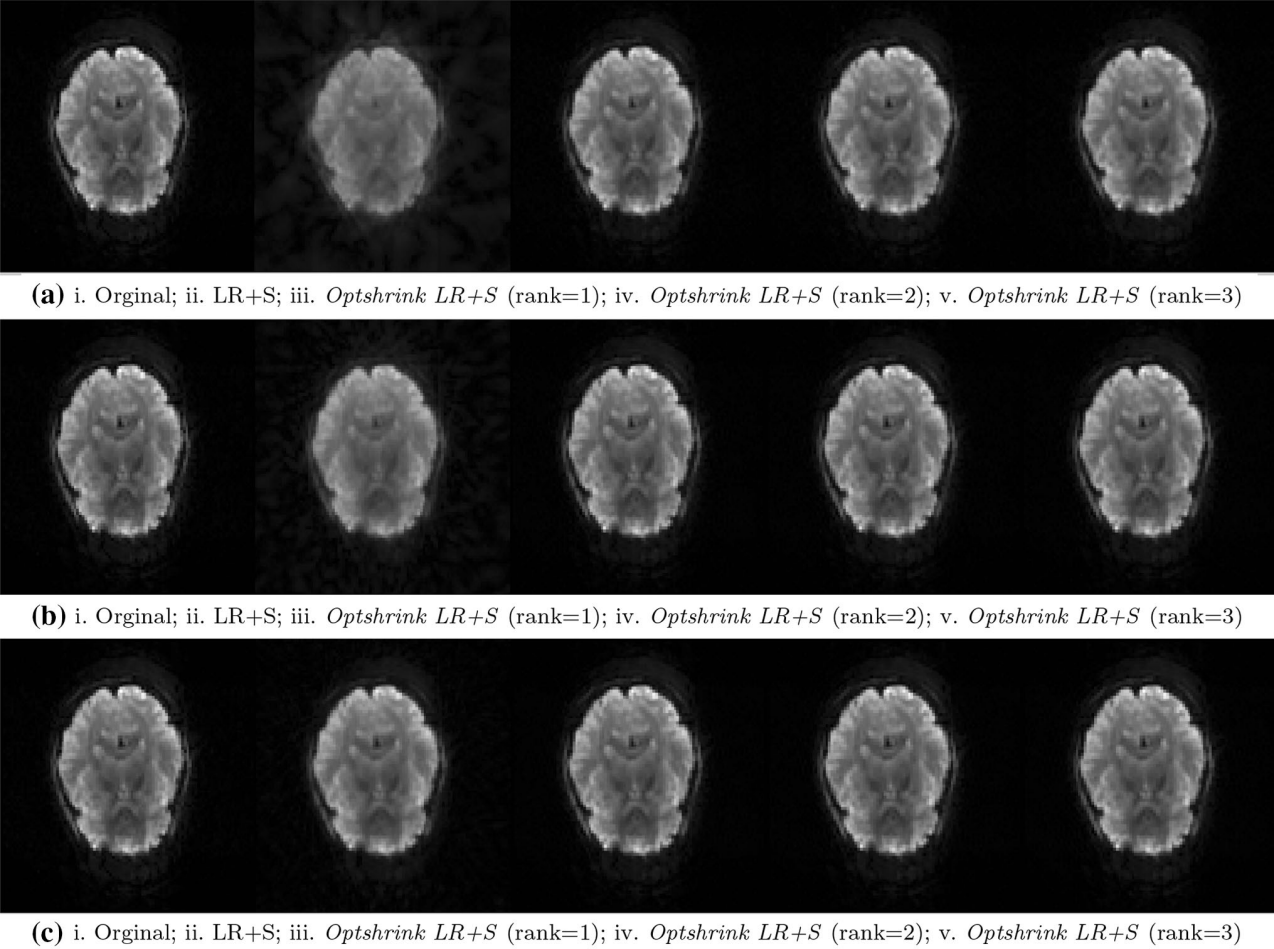
Resting-state fMRI data—original and reconstructed slice no. 24 [*left to right* Original; LR + S; *Optshrink LR + S* (rank = 1); *Optshrink LR + S* (rank = 2); *Optshrink LR + S* (rank = 3)]: **a** 6 radial lines (time point 100); **b** 12 radial lines (time point 100); **c** 24 radial lines (time point 100)

Likewise, Fig. 4 shows one of the reconstructed brain slices of resting-state dataset (Baltimore dataset) using the proposed *Optshrink LR + S* and the existing LR + S [15] method. Reconstructed data are visually shown at different radial lines in Fig. 4a, b, c corresponding to the middle slice (slice number 24 of 47 no. of total slices) captured at the 100th time point.

Following observations can be drawn from the reconstructed slices of both task-based and resting-state data shown in Figs. 3 and 4:

1. Slices reconstructed using LR + S method show a decline in quality with decrease in the number of radial sampling lines. On the other hand, reconstruction results with the proposed *Optshrink LR + S* method are quite consistent and the reconstruction quality does not fall by a great deal with the reduction in number of sampling lines.
2. Slices reconstructed using different radial sampling patterns consistently show that LR + S method output is blurred and the slices have artifacts at the center and the boundary compared to the proposed *Optshrink LR + S*. This observation indicates that there is SNR loss with LR + S method that may lead to incorrect brain activation detection. On the other hand, slices reconstructed using the proposed *Optshrink LR + S* method are very clear and free of artifacts.
3. Reconstruction using *Optshrink LR + S* method is robust to input rank specification. Hence, rank definition is not a bottleneck in the proposed *Optshrink LR + S*.

All the above observations indicate that we can reconstruct fMRI data with greater quality by sampling much lesser measurements in *k*–*t* space with the proposed *Optshrink LR + S* method compared to the existing LR + S method. Hence, higher acceleration rate is possible with *Optshrink LR + S* method.

**Fig. 5:**
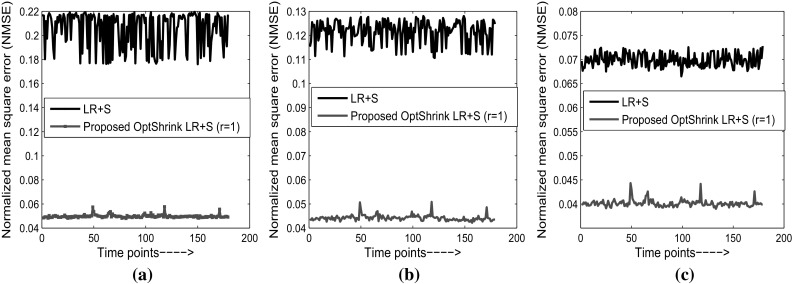
Normalized mean square error versus time points on task-based fMRI dataset (slice no. 18): **a** 6 radial lines (12.856 acceleration factor); **b** 12 radial lines (6.065 acceleration factor); **c** 24 radial lines (3.495 acceleration factor)

**Fig. 6:**
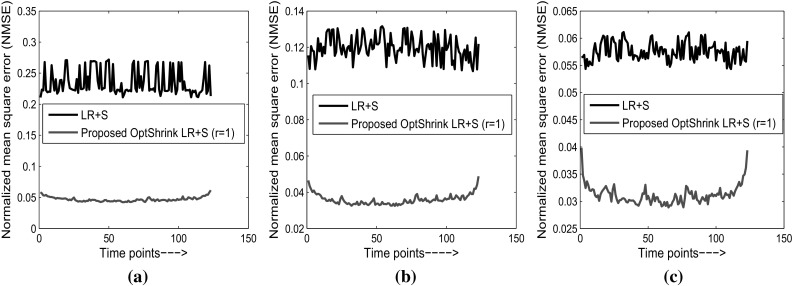
Normalized mean square error versus time points on resting-state fMRI dataset (slice no. 24): **a** 6 radial lines (12.856 acceleration factor); **b** 12 radial lines (6.065 acceleration factor); **c** 24 radial lines (3.495 acceleration factor)

Figures 5 and 6 show quantitative results via normalized mean square error (NMSE) versus time for both the dataset with different radial sampling patterns where:

19 $$\begin{aligned} \hbox {NMSE}=\left\| {\mathbf {I}}-\hat{\mathbf {I}} \right\| _{2}/\left\| {\mathbf {I}} \right\| _{2}, \end{aligned}$$

$||\cdot ||_2$ denotes $l^2$ norm and, ${\mathbf {I}}$ and $\hat{\mathbf {I}}$ are the original and reconstructed brain slices, respectively. NMSE values are computed between reconstructed and original slice at each time point. We represent reconstructed results using LR + S method and *Optshrink LR + S* method. In consonance with the qualitative results of Figs. 3 and 4, we observe higher NMSE with LR + S method compared to the proposed *Optshrink LR + S* method. While the NMSE increases rapidly with decrease in radial lines with LR + S method, it remains quite consistent with *Optshrink LR + S* method. In order to assess other reconstruction methods quantitatively, we present reconstruction results in Table 1 in terms of NMSE and peak signal-to-noise ratio (PSNR) on both the datasets. From Table 1, we note that NMSE increases with decrease in the number of radial lines, i.e., with fewer *k*-space measurements with existing reconstruction methods. On the other hand, the proposed *Optshrink LR + S* method shows consistent PSNR and NMSE values at different radial lines.

Moreover, *Optshrink LR + S* results are similar with different input rank specification. These results are in line with the qualitative results observed with the reconstructed slice quality in Figs. 3 and 4. This is to note that the proposed *Optshrink LR + S* method estimates a denoised version of low-rank matrix and hence yields better results.

**Fig. 7:**
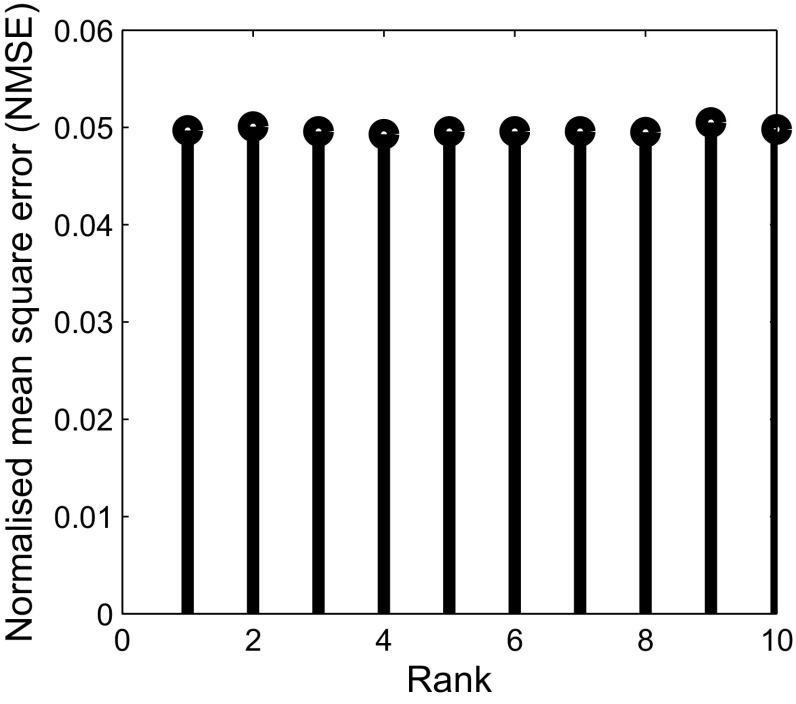
Task-based fMRI dataset, slice no. 18, time point 100: NMSE versus rank of the proposed *Optshrink LR + S* method using 6 radial lines

**Fig. 8:**
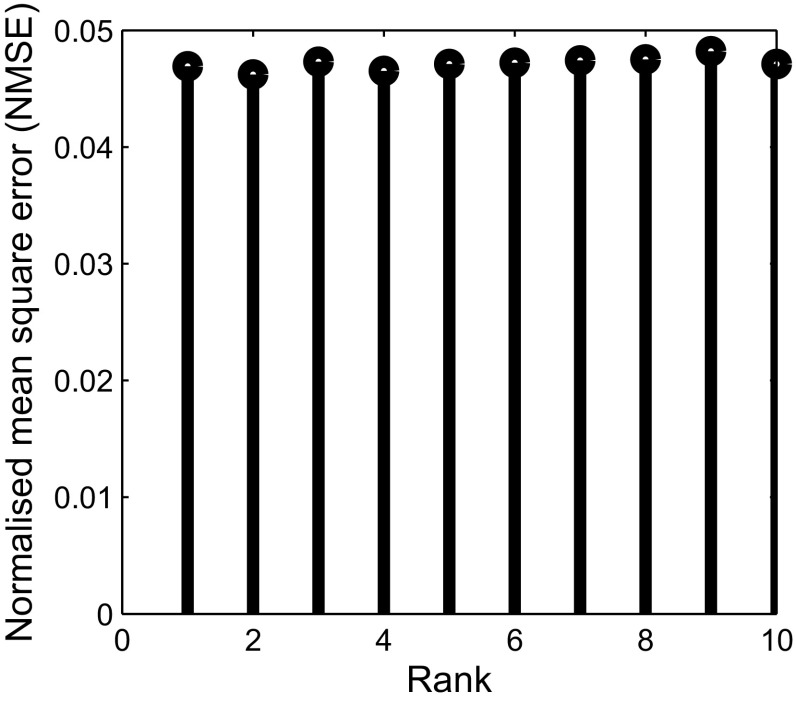
Resting-state fMRI dataset—slice no. 24, time point 100: NMSE versus rank of the proposed *Optshrink LR + S* method using 6 radial lines

In order to further ascertain the robustness of *Optshrink LR + S* method to rank, we present NMSE versus rank and PSNR versus rank for both the dataset in Figs. 7 and 8. We use 6 radial lines for undersampling *k*-space data and provide different rank as input to *Optshrink LR + S* method. The reconstruction accuracy remains similar for different rank values, and hence, any rough estimate of rank may be provided as input to this method for fMRI signal reconstruction.

### Group-level analysis

**Fig. 9:**
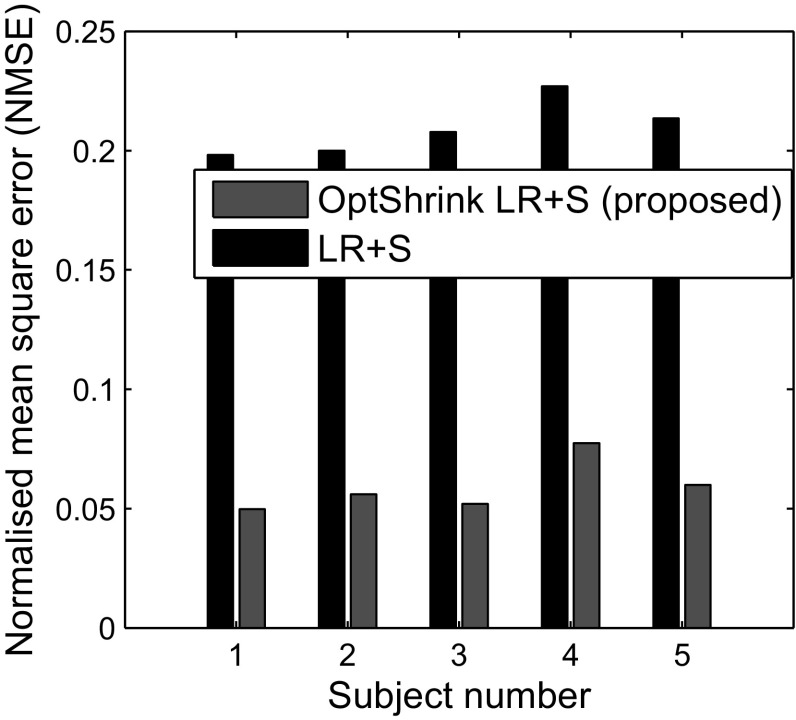
Task-based fMRI dataset—slice no. 18: NMSE versus subject number with the proposed *Optshrink LR + S* method (rank = 1) using 6 radial lines

**Fig. 10:**
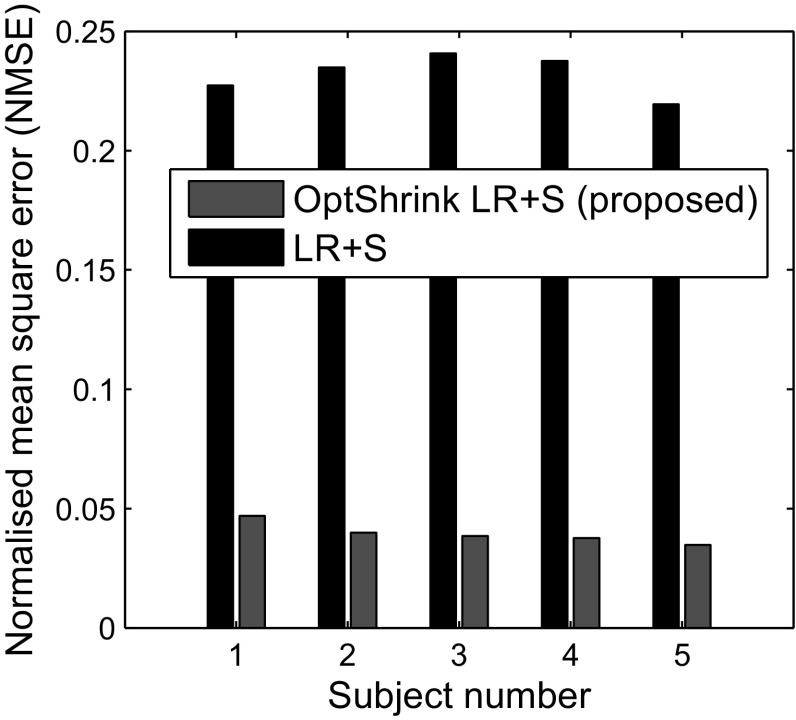
Resting-state fMRI dataset—slice no. 24: NMSE versus subject number with the proposed *Optshrink LR + S* method (rank = 1) using 6 radial lines

In Figs. 9 and 10, we present NMSE and PSNR results for five subjects each of task-based dataset and resting-state dataset, respectively. We use 6 radial lines for undersampling the *k*-space data. These results indicate that our proposed *Optshrink LR + S* is robust to subject variability and are reproducible across subjects.

**Table 1:** Reconstruction results with different methods on two datasets

Dataset	Method	NMSE			PSNR		
		6 lines	12 lines	24 lines	6 lines	12 lines	24 lines
Task-based dataset	Direct IFT	0.3088	0.2177	0.1595	3.54	6.58	9.63
	CS with wavelet sparsity [13]	0.2435	0.219	0.138	4.828	7.79	12.07
	LR + S [15]	0.1992	0.1215	0.0699	6.583	10.87	15.67
	Proposed Optshrink LR + S (*r* = 1)	0.0497	0.0442	0.0401	18.62	19.65	20.49
	Proposed Optshrink LR + S (*r* = 2)	0.0501	0.0437	0.0401	18.571	19.76	20.49
	Proposed Optshrink LR + S (*r* = 3)	0.0496	0.0435	0.0401	18.649	19.78	20.49
Resting-state dataset	Direct IFT	0.4067	0.286	0.1979	2.93	6.09	9.415
	CS with wavelet sparsity [13]	0.2917	0.2054	0.1193	5.42	8.465	13.19
	LR + S [15]	0.2351	0.1198	0.0576	7.321	13.16	19.52
	Proposed Optshrink LR + S (*r* = 1)	0.0469	0.0359	0.031	21.32	23.64	24.91
	Proposed Optshrink LR + S (*r* = 2)	0.0462	0.036	0.0311	21.44	23.62	24.88
	Proposed Optshrink LR + S (*r* = 3)	0.0473	0.0364	0.0312	21.24	23.52	24.85

Task-based data—false belief task fMRI data, subject no. 1, results on slice number 18, averaged over all time points

Resting-state data—Baltimore fMRI data, subject no. 1, results on slice number 24, averaged over all time points

### Subject-level statistical analysis on activation maps

In this section, we would like to study the effectiveness of *Optshrink LR + S* method with reference to brain activation detection. To this end, reconstruction is performed on the task-based dataset (false belief task) using LR + S method and *Optshrink LR + S* (rank = 1) method. Preprocessing of the original and the reconstructed fMRI dataset are performed using SPM12.^3^ We performed motion correction that is used to suppress motion-related artifacts. In general, motion correction is followed by smoothing as a preprocessing step so that the noise is Gaussian-distributed (by Central Limit Theorem). This establishes the validity of statistical tests using general linear model (GLM)-based analysis, a univariate approach, used for detecting brain activation in task-based fMRI data [45]. Since *Optshrink LR + S* method is supposed to provide denoised low-rank matrix, we tested the robustness of the proposed method on brain activation detection both with and without smoothing in the preprocessing pipeline.

**Fig. 11:**
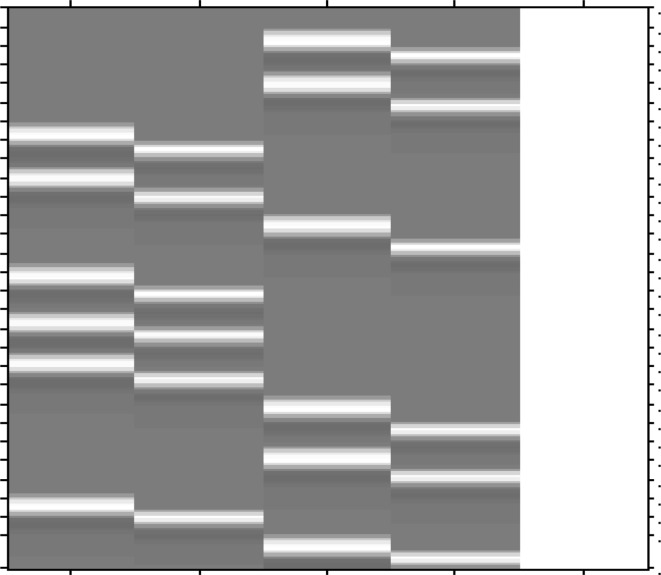
Design matrix of task-based fMRI dataset (false belief task)

In GLM, linear model is fitted to each voxel time series using the design matrix corresponding to the task stimulus. The estimated parameters are used to build statistical parametric maps (SPMs) [46]. Figure 11 shows the design matrix for the false belief dataset that consists of five conditions. First four conditions correspond to four different block stimuli (false belief story, false belief question, false photograph story, false photograph question [38]) that are convolved with the canonical hemodynamic response function (HRF) and form first four columns of the design matrix. The last column captures the linear trend of data.

**Fig. 12:**
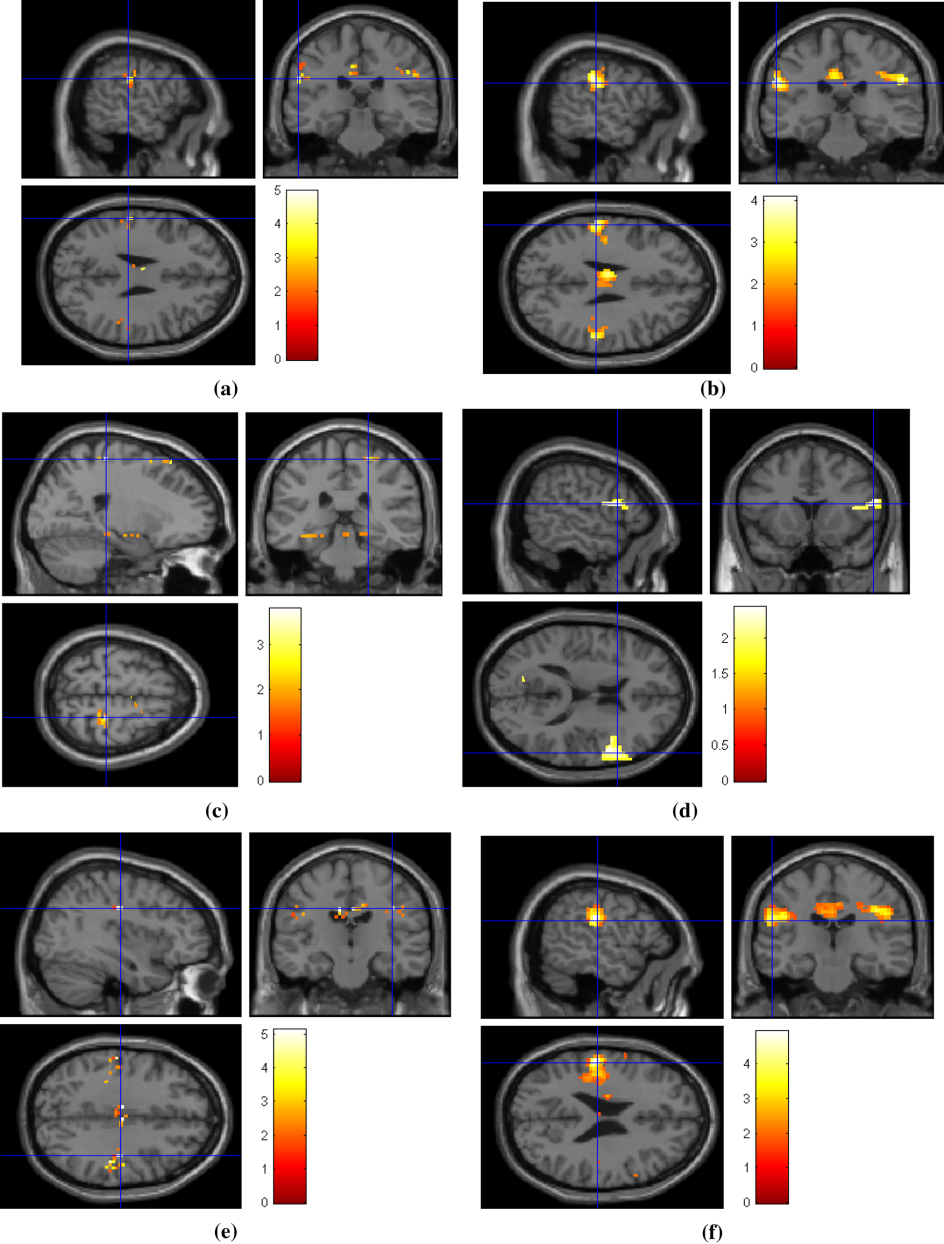
False belief fMRI data shown on sagittal, coronal, and axial planes: **a** fully sampled fMRI data; **b** smoothed fully sampled fMRI data; **c** reconstructed fMRI data using LR + S (6 radial lines) (without smoothing); **d** reconstructed fMRI data using LR + S (6 radial lines) (with smoothing); **e** reconstructed fMRI data using proposed *Optshrink LR + S* method (rank = 1) (6 radial lines) (without smoothing); **f** reconstructed fMRI data using proposed *Optshrink LR + S* method (rank = 1) (6 radial lines) (with smoothing)

**Fig. 13:**
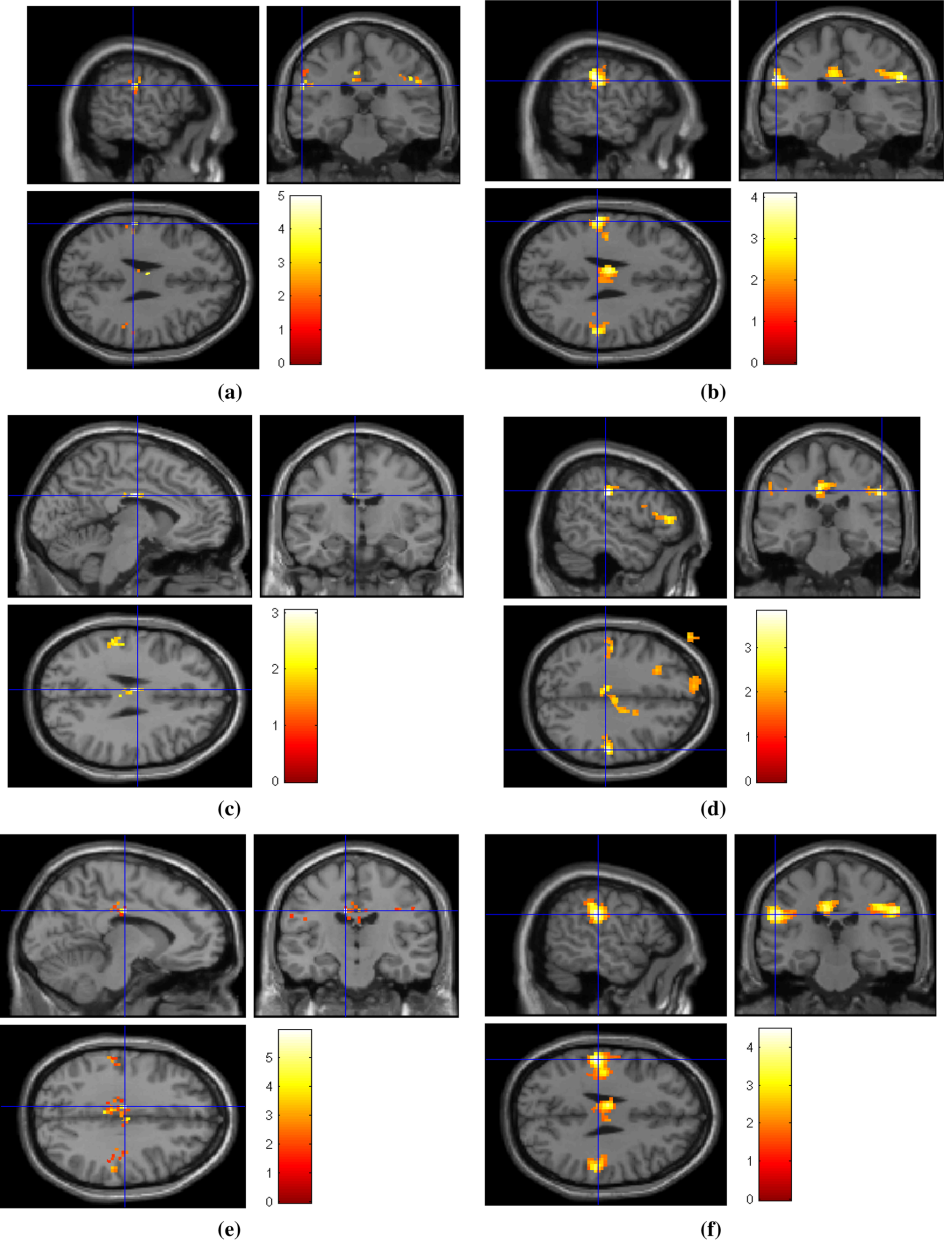
False belief fMRI data shown on sagittal, coronal, and axial planes: **a** fully sampled fMRI data; **b** smoothed fully sampled fMRI data; **c** reconstructed fMRI data using LR + S (12 radial lines) (without smoothing); **d** reconstructed fMRI data using LR + S (12 radial lines) (with smoothing); **e** reconstructed fMRI data using proposed *Optshrink LR + S* method (rank = 1) (12 radial lines) (without smoothing); (*f*) reconstructed fMRI data using proposed *Optshrink LR + S* method (rank = 1) (12 radial lines) (with smoothing)

**Fig. 14:**
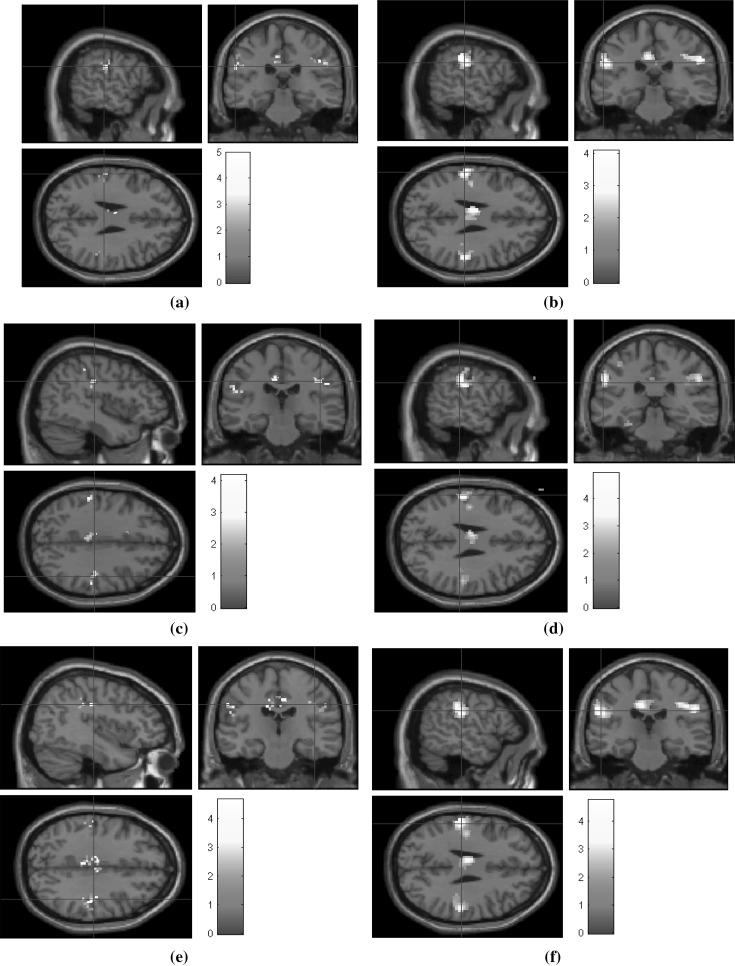
False belief fMRI data shown on sagittal, coronal, and axial planes: **a** fully sampled fMRI data; **b** smoothed fully sampled fMRI data; **c** reconstructed fMRI data using LR + S (24 radial lines) (without smoothing); **d** reconstructed fMRI data using LR + S (24 radial lines) (with smoothing); **e** reconstructed fMRI data using proposed *Optshrink LR + S* method (rank = 1) (24 radial lines) (without smoothing); **f** reconstructed fMRI data using proposed *Optshrink LR + S* method (rank = 1) (24 radial lines) (with smoothing)

Reconstruction is performed on undersampled fMRI data on three radial sampling patterns of 6, 12, and 24 radial lines. Figures 12,  13, and 14 show the corresponding statistical maps obtained using (a) original fully sampled *k*–*t* space data without smoothing operation (b) original smoothed fully sampled data, (c) reconstructed data using LR + S method (without smoothing), (d) reconstructed data using LR + S method (with smoothing), (e) reconstructed data using the proposed *Optshrink LR + S* method (without smoothing), and (f) reconstructed data using the proposed *Optshrink LR + S* method (with smoothing). We present results on representative slices having peak voxel of activation, whereas Montreal Neurological Institute (MNI) position of this most active voxel is listed in Table 2. We also report cluster sizes and maximum *z*-scores values in this table. Activation maps are thresholded t test at cluster level with uncorrected *p* value = 0.05. Clusters with less than 12 voxels are rejected.

**Table 2:** Statistical analysis results for uncorrected

	Method	6 lines			12 lines			24 lines		
		Cluster size	*Z* score	MNI position	Cluster size	*Z* score	MNI position	Cluster size	*Z* score	MNI position
1	LR + S [15] (without smoothing)	12	3.7	23 −31 62	14	3	−6 −13 29	36	4.06	45 −24 34
2	LR + S [15] (with smoothing)	93	2.41	58 14 15	50	3.74	54 −24 35	122	4.56	−57 −30 28
3	Proposed Optshrink LR + S (*r* = 1) (without smoothing)	35	4.94	39 −20 34	57	5.64	−9 −16 33	42	4.52	42 −23 34
4	Proposed Optshrink LR + S (*r* = 1) [with smoothing (FWHM = 6 mm)]	218	4.93	−54 −24 24	204	4.34	−54 −27 28	162	4.6	−54 −27 28

Task-based data—false belief task fMRI data, subject no. 1

Fully sampled fMRI data—cluster size = 26, *Z* score = 4.79, MNI position (in mm) = −57 −27 28

Smoothed fully sampled fMRI data with FWHM = 6 mm—cluster size = 112, *Z* score = 3.99, MNI position (in mm) = −57 −27 28

Please note that the coordinates of most active voxel are reported via *Z* score. Cluster size denotes the number of active voxels surrounding this most active voxel

Brain activation maps using original fully sampled smoothed data show better results compared to the original fully sampled data without smoothing [compare (b) with (a) in Figs. 12, 13, 14]. As evident from Figs. 12, 13, 14 and Table 2, we notice that LR + S method provides inferior results, while activation maps using data reconstructed with *Optshrink LR + S* method (with smoothing in preprocessing) provides activation maps similar to those of (b). The MNI position of the most active voxel on the reconstructed data using the proposed Optshrink LR + S method (with smoothing) is same as that obtained with the original data. Smoothing helps in increasing the sensitivity of BOLD time series. It can be noticed via increase in cluster size containing active voxels. Original smoothed fMRI data cluster size is 112 while without smoothed cluster size is 26. In the case of the proposed Optshrink LR + S method (with smoothing), the cluster size of reconstructed data is 204. Also, these clusters of activation maps are consistently good at all acceleration factors. This clearly shows that the proposed method provides enhanced brain activation maps and is indeed better compared to other reconstruction methods.

### Reproducibility of resting-state networks

In this section, we test the efficacy of the proposed Optshrink LR + S method on resting-state fMRI dataset. We evaluate performance in terms of reproducibility of resting-state networks (RSNs). We compare RSNs of Optshrink LR + S-based reconstructed data with the RSNs obtained from the fully sampled original fMRI data that is considered as the ground truth. RSNs are identified using the spatial independent component analysis (ICA) of GIFT toolbox.^4^

Before ICA is applied, data are preprocessed. The first five fMRI brain volumes are discarded followed by slice-time correction. Next, realignment is done for motion correction followed by spatial normalization onto the Montreal Neurological Institute (MNI) space (3-mm isotropic voxels). In the end, brain volumes are spatially smoothed with a Gaussian kernel [full width half maximum (FWHM) = 6 mm].

After preprocessing, we utilize InfomaxICA algorithm in GIFT to obtain 100 independent spatial components. We identified 54 RSNs from mean maps of all five fully sampled ground truth fMRI data (corresponding to five subjects) after removing artifact components. These RSNs can be broadly categorized into 10 RSNs: (1) Visual network (VN), (2) Somatomotor network (SMN), (3) Limbic network (LN), (4) Dorsal attention network (DAN), (5) Ventral attention network (VAN), (6) Default mode network (DMN), (7) Frontoparietal network (FPN), (8) Temporal + Frontal network (TFN), (9) Subcortical network (SCN), and (10) Cerebellar network (CN). We also ran ICA on the reconstructed data. These dataset are reconstructed using 16.49% (12 radial lines) acquired samples in *k*-space using Optshrink LR + S with rank one. We identified 56 RSNs from mean spatial components. These RSNs can be further classified into various categories as mentioned above.

**Fig. 15:**
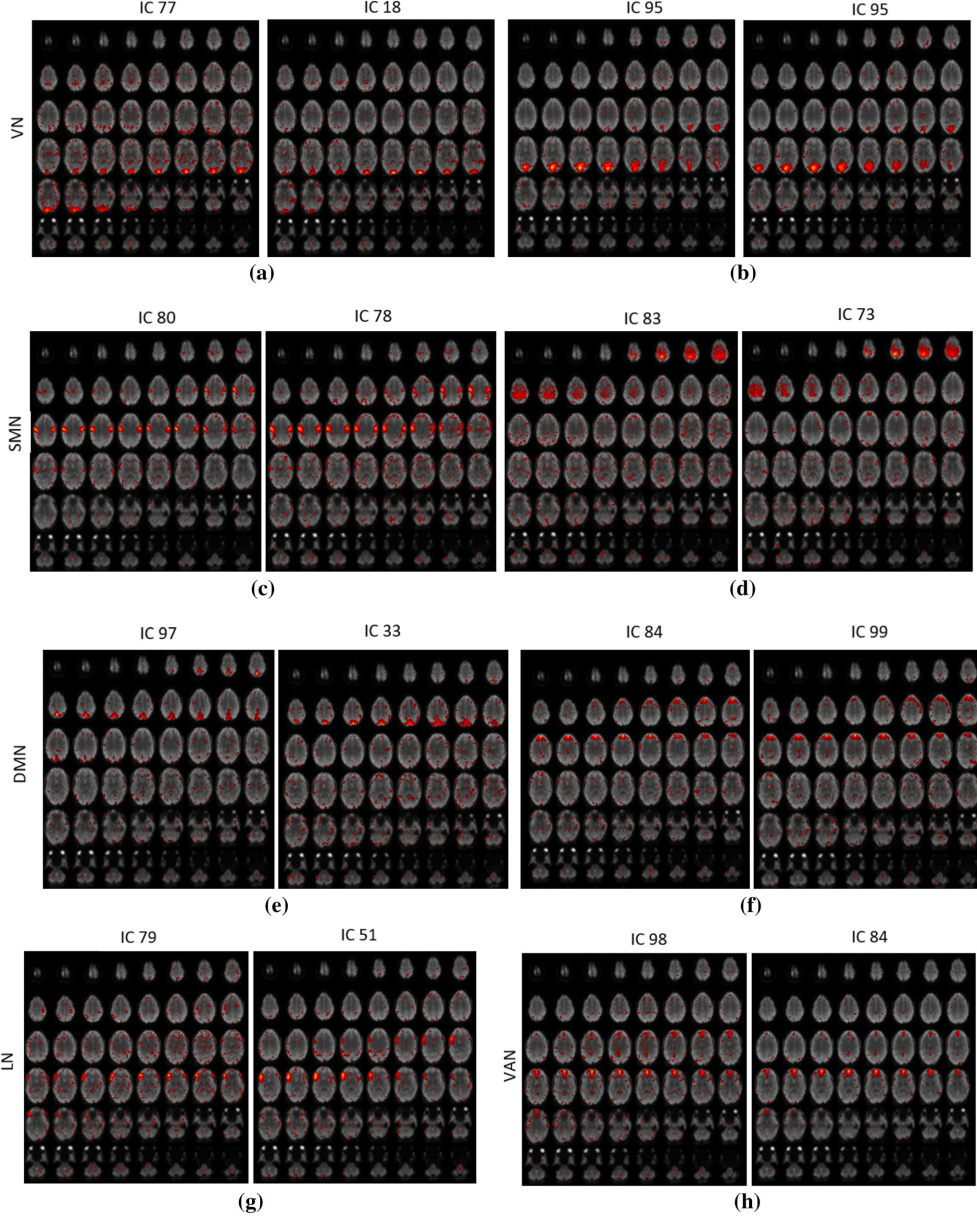
Axial view of spatial maps of various RSNs where *left part* of each figure is from the original fully available dataset and *right part* is from the Optshrink LR + S reconstructed data

**Fig. 16:**
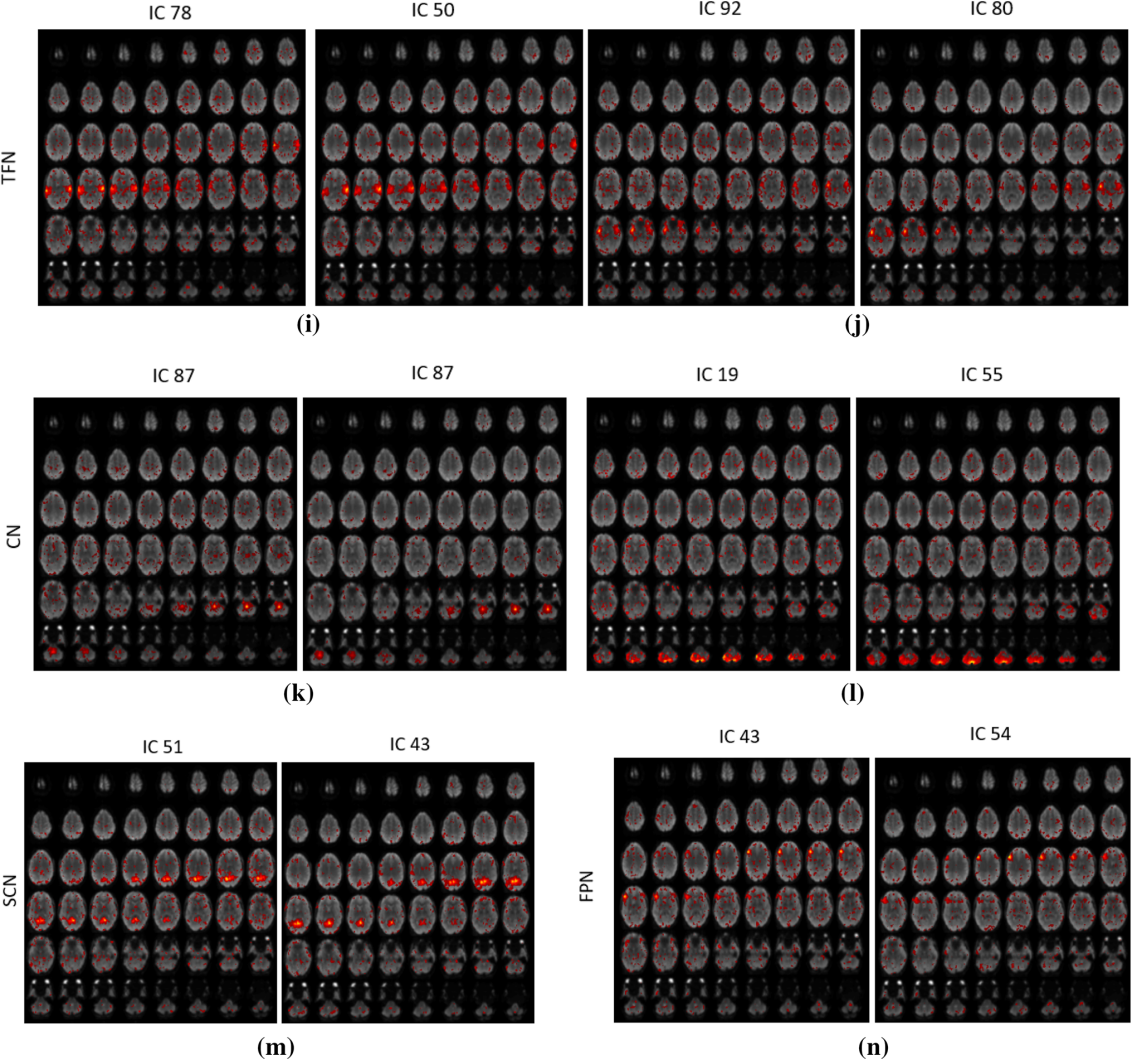
Axial view of spatial maps of various RSNs where *left part* of each figure is from the original fully available dataset and *right part* is from the Optshrink LR + S reconstructed data

Figures 15 and 16 show some of the RSNs obtained from fully sampled ground truth data and Optshrink LR + S reconstructed data. From this figure, we observe that RSNs identified by Optshrink LR + S reconstructed data resemble with the ground truth fully sampled data. This shows that Optshrink LR + S method is able to preserve functional characteristics of data. This is the most desirable need in neuroimaging research. Please note that we also ran ICA on reconstructed data using LR + S. We observed more artifact components with a total 100 spatial components. This again validates our claim that the proposed Optshrink LR + S method is working better than existing methods in the literature.

## Conclusion

In this paper, we have proposed a new accelerated fMRI method, named *Optshrink LR + S* method, for fMRI reconstruction from undersampled *k*–*t* space data. The proposed method exploits sparsity and low-rank decomposition with denoising to improve fMRI reconstruction accuracy. Comparison results demonstrate that the reconstruction performance of the proposed *Optshrink LR + S* method is superior to existing methods at various acceleration factors. While the performance of the existing methods falls rapidly at faster acceleration rates, Optshrink LR + S method performs consistently. Quantitative and qualitative results, group-level and subject-level analyses, show the superior performance of the proposed method. In addition, *Optshrink LR + S* method provides enhanced brain activation maps that is an added but most useful advantage of the proposed method. MATLAB implementation of proposed algorithm is available online.^5^

## Footnotes

1
https://openfmri.org/dataset/.

2
http://www.nitrc.org/frs/?group_id=296

3
http://www.fil.ion.ucl.ac.uk/spm/

4
https://www.nitrc.org/projects/gift.

5
http://in.mathworks.com/matlabcentral/fileexchange/60836-optshrink-lr+s--accelerated-fmri-reconstruction-using-non-convex-optimal-singular-value-shrinkage.
